# 10-Year cardiovascular event risks for women who experienced hypertensive disorders in late pregnancy: the HyRAS study

**DOI:** 10.1186/1471-2393-10-28

**Published:** 2010-06-01

**Authors:** Wietske Hermes, Arie Franx, Maria G van Pampus, Kitty W Bloemenkamp, Joris A van der Post, Martina Porath, Gabrielle Ponjee, Jouke T Tamsma, Ben W Mol, Christianne J de Groot

**Affiliations:** 1 Department of Obstetrics and Gynecology, Medical Centre Haaglanden Den Haag, the Netherlands; 2 Department of Obstetrics and Gynecology, Leiden University Medical Centre, the Netherlands; 3 Department of Obstetrics and Gynecology, Sint Elisabeth Hospital Tilburg, the Netherlands; 4 Department of Obstetrics and Gynecology, University Medical Centre Groningen, the Netherlands; 5 Department of Obstetrics and Gynecology, Academic Medical Centre Amsterdam, the Netherlands; 6 Department of Obstetrics and Gynecology, Maxima Medical Centre Veldhoven, the Netherlands; 7 Department of Clinical Laboratory, Medical Centre Haaglanden Den Haag, the Netherlands; 8 Department of Internal Medicine, Leiden University Medical Centre, the Netherlands

## Abstract

**Background:**

Cardiovascular disease is the cause of death in 32% of women in the Netherlands. Prediction of an individual's risk for cardiovascular disease is difficult, in particular in younger women due to low sensitive and specific tests for these women. 10% to 15% of all pregnancies are complicated by hypertensive disorders, the vast majority of which develop only after 36 weeks of gestation. Preeclampsia and cardiovascular disease in later life show both features of "the metabolic syndrome" and atherosclerosis. Hypertensive disorders in pregnancy and cardiovascular disease may develop by common pathophysiologic pathways initiated by similar vascular risk factors. Vascular damage occurring during preeclampsia or gestational hypertension may contribute to the development of future cardiovascular disease, or is already present before pregnancy. At present clinicians do not systematically aim at the possible cardiovascular consequences in later life after a hypertensive pregnancy disorder at term. However, screening for risk factors after preeclampsia or gestational hypertension at term may give insight into an individual's cardiovascular risk profile.

**Methods/Design:**

Women with a history of preeclampsia or gestational hypertension will be invited to participate in a cohort study 2 1/2 years after delivery. Participants will be screened for established modifiable cardiovascular risk indicators. The primary outcome is the 10-year cardiovascular event risk. Secondary outcomes include differences in cardiovascular parameters, SNP's in glucose metabolism, and neonatal outcome.

**Discussion:**

This study will provide evidence on the potential health gains of a modifiable cardiovascular risk factor screening program for women whose pregnancy was complicated by hypertension or preeclampsia. The calculation of individual 10-year cardiovascular event risks will allow identification of those women who will benefit from primary prevention by tailored interventions, at a relatively young age.

**Trial registration:**

The HYPITAT trial is registered in the clinical trial register as ISRCTN08132825.

## Background

Cardiovascular disease is the cause of death of 32% of women in the Netherlands [1]. Not all women are at the same risk of cardiovascular disease. Prediction of risk in younger women is particularly difficult due to low sensitive and specific tests for these women. Identification of individual women at higher risk is a challenge. This proposal takes an innovative angle to gain insight in cardiovascular morbidity and mortality later in life in women using pregnancy related hypertensive complications. Approximately 10% to 15% of all pregnancies are complicated by hypertension and largely contribute to maternal and neonatal morbidity and mortality worldwide. In the Netherlands it is the largest single cause of maternal mortality [2]. The vast majority of hypertensive disorders present themselves after 36 weeks of gestation [3]. Therefore, we focus in this study on those women with pregnancy related hypertensive complications (near) at term (> 36 weeks gestation).

The etiology of hypertensive disorders of pregnancy is not fully understood, but the causal treatment is delivery of the baby and the placenta. Recently it was shown that in women at term with gestational hypertension or preeclampsia induction of labor is advisable to avoid progression to more severe disease [4]. However, the health status of these women after pregnancy has been given little of any attention in routine clinical practice up to now. Obstetricians and midwifes are traditionally completely focused on pregnancy outcome and do not seem to bother about the significance of complications of pregnancy for the future health of the mother, this is also true for general practitioners.

Recently, data from epidemiologic studies incited the novel concept of pregnancy as cardiovascular challenge test; women who have had a pregnancy complicated by hypertensive disorders are prone to develop cardiovascular disease in later life [5-10]. In line with this concept is that pregnancy acts as a metabolic and cardiovascular stress test for the mother. During pregnancy a failure to meet the physiological demands will unmask impaired organ function, e.g. hypertension will arise and most often subside after delivery. However, these failures will remanifest in later life when the cumulative effects of ageing diminish the reserves of an already vulnerable (organ) system [5]. Jonsdottir et al. examined causes of death in 374 women with a history of hypertensive complications in pregnancy and noted that their death rate from complications of coronary heart disease was significantly higher than expected from analysis of population data [11].

This concept is further supported by case-control studies by others and ourselves, demonstrating that women with a history of early preeclampsia have higher circulating concentrations of fasting insulin, lipid and coagulations factors post partum than controls matched for body mass index [12,13]. These changes in vascular risk markers in women with a history of preeclampsia are part of the spectrum of the metabolic syndrome. The metabolic syndrome is hypothesised to be a key factor underlying cardiovascular disease and in particular coronary heart disease.

The mechanism of the link between preeclampsia and cardiovascular disease has not been clarified. Hypertensive disorders in pregnancy and cardiovascular disease may develop by common pathophysiologic pathways initiated by similar risk factors. Permanent vascular damage may occur during preeclampsia or gestational hypertension and subsequently contributes to the development of cardiovascular disease in later life, or cardiovascular disease is already present before pregnancy. However, determinants or risk indicators to be measured after pregnancy which would predict cardiovascular disease are lacking. Such risk indicators may identify women at risk at an early stage for them to benefit from intervention.

The concept described above is based on studies focusing on early, severe preeclampsia, which is a relatively rare disorder [14], while preeclampsia at (near) term is mostly mild and more common (75% of cases) [15]. Therefore, prospective evaluation of those women is required to identify cardiovascular risk indicators after hypertensive pregnancy complications at term, with the eventual aim to offer these women the opportunity for primary prevention at a relatively young age [16].

We propose a cohort study to establish whether women with gestational hypertension or preeclampsia at term are at increased risk for cardiovascular disease in later life, and if these women are likely to benefit from tailored preventative interventions directed at modifiable cardiovascular risk indicators at a relative young age.

## Methods/Design

### Aims

The aim of this study is to identify modifiable risk indicators for future cardiovascular disease 2 1/2 years postpartum in women with (near) term preeclampsia or gestational hypertension. The proposed research concerns a multi-centre cohort study in women who had a pregnancy complicated by mild preeclampsia or gestational hypertension (near) at term which is recently published [4]. This study will provide insight on the health costs and benefits of a screening program for all women with hypertensive complications at term. This study is embedded in a Dutch Obstetric Consortium in the Netherlands. Almost all obstetric centres nationwide participate in this structure, including academic hospitals, non-academic teaching hospitals and non-teaching hospitals.

### Participants: Exposed

In this study women who had preeclampsia or gestational hypertension at term, who participated in the HYPITAT study, will be eligible for the follow up study 2 1/2 years after their delivery. The HYPITAT study was a national randomised clinical trial in which women 18 years of age or older with gestational hypertension or mild preeclampsia at (near) term were included. Eligible were women with a singleton pregnancy in cephalic presentation and a gestational age between 36^+0 ^and 41^+0 ^weeks, whose pregnancy was complicated by gestational hypertension or mild preeclampsia. Gestational hypertension was defined as diastolic blood pressure equal to or above 95 mmHg measured at two occasions at least six hours apart in a woman who was normotensive at the start of pregnancy until week 20 of gestational age. Mild preeclampsia was defined as diastolic blood pressure equal to or above 90 mmHg measured at two occasions at least six hours apart combined with proteinuria. Proteinuria was defined as ≥ 2+ protein on dipstick, > 300 mg total protein in a 24 hour urine collection and/or protein/creatinine ratio > 30 mg/mmol. Exclusion criteria were severe gestational hypertension or severe preeclampsia, defined as diastolic blood pressure ≥ 110 mmHg, systolic blood pressure ≥ 170 mmHg and/or proteinuria ≥ 5 gram in 24 hours, pre-existing hypertension treated with antihypertensive drugs, diabetes mellitus, diabetes gravidarum requiring insulin therapy, renal disease, heart disease, previous caesarean section, HELLP syndrome, oliguria < 500 milliliter in 24 hours, pulmonary edema or cyanosis, HIV seropositivity, use of intravenous anti-hypertensive medication, fetal anomalies, intra-uterine growth restriction and abnormalities at the fetal heart rate (FHR) -monitoring. Participants were randomly allocated to induction of labour or expectant monitoring (n = 1162) [4].

Women at study entry (randomisation) were inquired for a follow up 2 years after delivery, the hypertension risk assessment study (HyRAS).

### Participants: Non-exposed

All exposed women will be asked to invite a friend as control. These women are required to have a history of one (or multiple) uncomplicated pregnancy (ies). This method will be used because of expected similar age and similar potential environmental exposures (i.e. socio-economic status). The non-exposed undergo the same procedure as the exposed at the local centre. Relatives of the exposed or of her partner will not be accepted in the non exposed group.

If the recruitment of non-exposed subjects in the method described above is not sufficient, the non-exposed group will be extended by approaching women with a history of at least one uncomplicated pregnancy in midwifery practices from three different locations in the Netherlands (Groningen, Leiden and The Hague).

### Ethical approval

The Hyras and HYPITAT studies were approved both primarily for all participating hospitals in The Netherlands by the medical ethics committee of Leiden University Medical Centre (HYPITAT: P04·210) and locally by the hospital board of the participating hospitals. The clinical trial registration number of the HYPITAT trial is: ISRCTN08132825.

### Procedures, recruitment and collection of baseline data

Women who participated in the HYPITAT study will be contacted by the research nurses 2 1/2 years after delivery (study entry date) and they will be invited to participate in the HyRAS study in their local centre. Before entry into the study the research nurse or midwife counsels the patients, asks informed consent and emphasizes that participation is voluntarily. Patients who decide not to participate in this study for complete follow up will be asked to fill out the questionnaire to be analyzed separately. This questionnaire includes medical history, psychological status, social status, use of medication, contraceptive methods, obstetric history, and family history including thrombosis, diabetes and cardiovascular disease. Furthermore, pregnancy and lactation are exclusion criteria's for risk factor screening and those women will be asked to only fill out the questionnaire and participate for risk factor screening (blood samples, urine collection and blood pressure, length, weight and anthropometrics measurement) 3 months after the delivery or lactation, with an extension to 12 months after the original study entry date.

After enrollment exposed and non-exposed subjects will be asked to fill out a questionnaire and will be invited for risk indicator screening (blood samples, urine collection and blood pressure, length, weight and anthropometrics measurement). Venous blood samples will be taken after an overnight fast for analysis of lipids and lipoproteins, insulin, glucose and high sensitive CRP. Urine will be collected immediately after waking up for microalbuminuria. After centrifuging the blood samples for 9 minutes at 3000 bpm at the local centre, all samples will be sent to the same laboratory (Medical Centre Haaglanden, the Hague, the Netherlands) and analyzed within 24 hours. Plasma will be prepared and stored at -70°C in 1.5 mL volumes until used. DNA will be isolated from leukocytes and stored at -20°C. Blood pressure will be measured manually in sitting position at the right upper arm. Body height (cm) and -weight (kg) will be measured with the participant dressed in light underclothes wearing shoes (except high heels) and waist circumference (cm) will be measured on uncovered skin using an inelastic tape measure with the participant in upright position, halfway between the rib cage and the pelvic bone. Hip circumference will also be measured.

Data- collection will be centralized and processed with adequate precautions to ensure patient confidentially. With the participant's informed consent, the study results will be sent to the general practitioner. Participants will also be informed on the results by mail, and in case of abnormal results it will be recommended to contact the general practitioner for evaluation or treatment.

### Outcome measures

#### Primary outcome measure

The primary outcome measure will be the 10-year cardiovascular event risk. An absolute 10-year cardiovascular risk (fatal and non-fatal) of > = 10% as estimated with the SCORE risk function, will be considered as elevated risk. In addition, risk will be calculated using the Adult Treatment Panel III risk score, Reynolds Risk Score and QRISK.

#### Secondary outcome measure

Secondary outcomes will be differences between exposed and non-exposed subjects in cardiovascular parameters, differences in SNP's in glucose metabolism, and neonatal outcome 2 1/2 years after pregnancy complicated by preeclampsia and gestational hypertension. Neonatal outcome includes neurological development and development of motor skills at the age of 2 1/2 years. We will use a parental questionnaire (the Child Behaviour Checklist (CBCL) and ages and stages questionnaire (ASQ)). The health costs and benefits of a screening program for women with hypertensive complications at term will be analyzed.

### Statistical issues

#### Sample size

Due to the young age of our participants, the estimated absolute 10-year cardiovascular risk is likely to be low. Therefore, the approach taken for each woman is to estimate the risk as if the woman was 60 years of age. This approach has been recommended in the cardiovascular risk factor management guidelines for young women with elevated risk factor levels. We plan to include women in 3:1 ratio, i.e. three women who had hypertension in pregnancy as compared to 1 control. A sample size of 414 women (310 exposed, 104 non exposed) is sufficient to demonstrate a risk increase from 5% to at least 15% (sided alpha .05. power 80%). This estimate is in line with our earlier studies showing a risk estimate > = 10% in over 30% of the *early *preeclampsia women [17].

#### Data analysis

All data are primarily analyzed to investigate the costs and potential health benefits of a screening program in order to assess if this screening programme is justified for all women with hypertensive pregnancy complications at term. In order to investigate the association between hypertensive disorders in pregnancy and maternal cardiovascular status Mann Whitney U tests or where indicated Chi-square will be used for comparisons between groups (exposed vs. non-exposed). Analysis in subgroups (i.e. gestational hypertension and preeclampsia) will be performed. Logistic regression analyses will be done with the maternal scores as dependent variables and the metabolic status, obstetric history, cardiovascular risk factors, diabetes risk factors and as independent variables.

## Discussion

Cardiovascular disease is the main cause of death of women in the Netherlands. In this study we will identify women that may be at higher risk for cardiovascular disease by their pregnancy complication: hypertensive pregnancy disorders at (near) term. Gestational hypertension and preeclampsia at (near) term are very common complications in pregnancy. Data concerning long term effects of pregnancies complicated by a hypertensive disorder at (near) term on cardiovascular disease are lacking for these women. Most studies concerning the effect of hypertensive disorders in pregnancy and cardiovascular disease in later life studied women who had severe *early-onset *preeclampsia, which is a rare disorder. Therefore, we conduct present study, which will provide evidence for the necessity of screening women on cardiovascular risk factors 2 1/2 years after a pregnancy complicated by *term *gestational hypertension or preeclampsia.

## Abbreviations

SNP: Single nucleotide polymorphism.

## Competing interests

The authors declare that they have no competing interests.

## Authors' contributions

CJMdG, AF, BWM were involved in conception and design of the study.

WH, CJMdG and AF drafted the manuscript. All authors mentioned in the manuscript are member of the HyRAS study group. They participated in the design of the study during several meetings and are local investigators at the participating centres. All authors edited the manuscript and read and approved the final manuscript.

## Pre-publication history

The pre-publication history for this paper can be accessed here:

http://www.biomedcentral.com/1471-2393/10/28/prepub
